# Balance Asymmetry in Parkinson’s Disease and Its Contribution to Freezing of Gait

**DOI:** 10.1371/journal.pone.0102493

**Published:** 2014-07-17

**Authors:** Tjitske A. Boonstra, Jeroen P. P. van Vugt, Herman van der Kooij, Bastiaan R. Bloem

**Affiliations:** 1 Department of Biomechanical Engineering, University of Twente, MIRA institute for biomechanical technology and technical medicine, Enschede, The Netherlands; 2 Department of Neurology, Medical Spectrum Twente, Enschede, The Netherlands; 3 Department of Biomechanical Engineering, Delft University of Technology, Delft, The Netherlands; 4 Radboud University Nijmegen Medical Centre, Department of Neurology, Donders Institute for Brain, Cognition and Behaviour, Nijmegen, The Netherlands; University Medical Center Groningen UMCG, Netherlands

## Abstract

Balance control (the ability to maintain an upright posture) is asymmetrically controlled in a proportion of patients with Parkinson’s disease. Gait asymmetries have been linked to the pathophysiology of freezing of gait. We speculate that asymmetries in balance could contribute to freezing by a) hampering the unloading of the stepping leg and/or b) leading to a preferred stance leg during gait, which then results in asymmetric gait. To investigate this, we examined the relationship between balance control and weight-bearing asymmetries and freezing. We included 20 human patients with Parkinson (tested OFF medication; nine freezers) and nine healthy controls. Balance was perturbed in the sagittal plane, using continuous multi-sine perturbations, applied by a motion platform and by a force at the sacrum. Applying closed-loop system identification techniques, relating the body sway angle to the joint torques of each leg separately, determined the relative contribution of each ankle and hip joint to the total amount of joint torque. We also calculated weight-bearing asymmetries. We determined the 99-percent confidence interval of weight-bearing and balance-control asymmetry using the responses of the healthy controls. Freezers did not have larger asymmetries in weight bearing (*p* = 0.85) nor more asymmetrical balance control compared to non-freezers (*p* = 0.25). The healthy linear one-to-one relationship between weight bearing and balance control was significantly different for freezers and non-freezers (*p* = 0.01). Specifically, non-freezers had a significant relationship between weight bearing and balance control (*p* = 0.02), whereas this relation was not significant for freezers (*p* = 0.15). Balance control is asymmetrical in most patients (about 75 percent) with Parkinson’s disease, but this asymmetry is not related to freezing. The relationship between weight bearing and balance control seems to be less pronounced in freezers, compared to healthy controls and non-freezers. However, this relationship should be investigated further in larger groups of patients.

## Introduction

Parkinson’s disease (PD) is typically an asymmetrical disease. The motor symptoms usually affect one side of the body first, and even though the contralateral side becomes involved later on, the initially affected side remains most prominently affected throughout the course of the disease in about 80 percent of patients [1], [2]. Asymmetries in balance control (i.e. when one leg is producing more force than the other leg in order to keep the body upright) have rarely been investigated. Pilot studies using posturography have shown that balance control – which is intuitively a very symmetrical task – can also be asymmetrically affected in PD [3], [4]. A study of balance control asymmetries in 17 PD patients (tested ON medication) showed that 24 percent of patients had a postural asymmetry [5]. This suggests that balance control can be asymmetrical in PD, but not in all individual patients.

Gait is also typically asymmetrically affected in PD [6]–[8]. Asymmetries in leg coordination have been linked to the pathophysiology of freezing of gait (FoG; [9], [10]). Specifically, PD patients with FoG (“freezers”) had more asymmetric leg swing times compared to PD patients who never experienced FoG (“non-freezers”). FoG is an episodic, disabling gait disorder during which the feet appear to be”glued to the floor” [11]. About 50 percent of patients with PD experience FoG, hence like balance asymmetry, this is not a consistent sign across all patients [12]. Interestingly, FoG most frequently occurs during tasks that require asymmetric motor control, such as turning while walking or when patients start walking [13], [14]. Gait initiation again involves motor asymmetry, because the step leg must be unloaded [14], thereby introducing an asymmetric medio-lateral weight distribution. Other indirect evidence for a possible relationship between asymmetric motor control and FoG comes from a study by Fasano and colleagues [15], who showed that asymmetrical deep-brain stimulation (i.e., stimulating the most affected side the most) reduced freezing episodes. Furthermore, recent studies showed that freezers exhibited asymmetric brain connectivity in the locomotor pathway [16], [17]. Moreover, recent studies in stroke patients show a relationship between asymmetric balance control and asymmetric gait [18].

Based on these observations, we speculated that asymmetries in balance (e.g., an asymmetric weight bearing) could prevent subjects from making an adequate weight shift that is needed to unload the stepping leg, and that this difficulty in weight shifting would in turn produce FoG. In addition, one could imagine that asymmetrical balance control (i.e., asymmetric joint torques) could lead to preference for a stance leg during gait (i.e., the better leg), leading to asymmetric gait as reported previously. When the gait pattern reaches a certain level of asymmetry, disordered bilateral coordination between the legs can occur (as was shown by this model study [19]), which could then lead to freezing [9]–[12].

Hence, we hypothesized that patients with FoG would have larger asymmetries in balance control compared to non-freezers. Such a possible relationship between asymmetric balance control and FoG has never been investigated.

To investigate this, we determined weight-bearing and balance-control asymmetries in a group of PD patients (tested OFF medication; both freezers and matched non-freezers), as well as healthy controls and related these to FoG, disease severity, walking difficulties, and history of falls.

## Methods

To investigate balance control asymmetries in PD patients, we approached the balance control system as a feedback system [20], [21]. That is, information about the position of the body is sent to the central nervous system (CNS), which determines a corrective action to keep the body upright. In such closed-loop systems, it is difficult to determine whether a movement occurs due to an external perturbation (such as the destabilizing effect of gravity) or is a result of a muscle contraction [22]. To “open” this loop, we applied mechanical perturbations and subsequently applied system- identification methods to determine the balance-control contribution of each leg separately (i.e., the exerted joint torque in response to body movement); the methods are described in detail elsewhere [21], [23]–[26]. However, it is important to note that the applied methods assume that the characteristics of the studied system do not change during the course of the experiment. This means that we instructed the participants not to change strategy (e.g., switch between responding stiff or slack) during the experiment, nor to take a step or swing their arms in response to the perturbations. Hence, we did not investigate actual freezing episodes, but examined steady-state behavior in freezers and non-freezers; comparable to other studies (e.g. [9], [16], [27]).

### Participants

We included 20 patients with PD (six female, nine with FoG, matched for disease severity with the 11 non-freezers, see Table 1) and nine matched healthy controls (two female; see Table 2). Patients were assessed in a practically defined OFF state, at least 12 hours after intake of their last dose of dopaminergic medication. Disease severity was determined using the Hoehn and Yahr stages and the motor part of the Unified Parkinson’s Disease Rating Scale [28]. Freezing of gait was quantified using the new freezing of gait questionnaire [29]. Patients were classified as freezers when they reported unequivocal subjective episodes of FoG (i.e. frequently experiencing the typical feeling of the feet being glued to the floor) during an interview with an experienced assessor. Non-freezers reported never having experienced freezing episodes. Furthermore, we provoked FoG by having the patients make fast and slow 360° turns toward the left and right body side [30]. Of the nine freezers, three of them showed freezing, while the non-freezers showed no freezing episodes during this test. Items 3.9–3.13 of the UPDRS were used to determine the Postural Instability and Gait Difficulty score. Clinical asymmetry was defined as a difference between the summed UPDRS scores of the left and right extremities (items 3.3–3.8 and 3.15–3.17). We asked about prior (near−) falls and about fear of falling. Fear of falling was also individually determined with the modified Falls Efficacy Scale [31]. In addition, the 10-meter walk test and the Timed-Up-and-Go-Test were administered to quantify gait and balance impairment. Lastly, we determined the preferred leg by assessing which leg was used when forced to take a step. We repeated this test three times, and the preferred leg was the leg that was used the most for stepping.

**Table 1:** Patient characteristics and clinical scores.

Patient	Age (yrs.)	Gender	Disease duration (yrs.)	H&Y	FOG	UPDRS III	PIGD	Clinical asymmetry	Fall risk	mFES	TUG (s)	TMW (m/s)
1	47	F	3	2	0	24	3	Left	1	8	9,5	1,23
2	54	F	3	2	0	11	0	Right	0	1	12,5	1,32
3	58	M	2,5	1	0	14	1	Left	0	2	9,4	1,38
4	67	M	7,0	2	0	34	2	Left	1	4	7,9	1,36
5	73	M	3,0	2	0	26	1	Right	0	6	10,9	1,25
6	79	F	4,5	2	0	36	4	Left	0	6	7,8	1,06
7	55	F	3,5	2	0	23	5	Left	0	5	10,4	1,10
8	77	F	4,0	2	0	30	4	Left	1	1	11	1,10
9	73	M	8	2	0	30	2	Left	1	3	7,9	1,38
10	61	M	1,5	1	0	15	1	Left	0	0	12,9	1,41
11	66	M	4,5	2	0	26	4	Left	0	2	9,5	1,05
12	67	M	7	3	1	57	6	Left	1	7	19	0,77
13	58	M	6	3	1	17	5	Right	1	2	11,7	1,18
14	64	F	15	2	1	35	8	Right	0	14	10,7	0,94
15	69	M	9,0	2	1	24	1	Right	0	8	10,4	1,20
16	57	M	3,0	2	1	39	1	Left	0	0	6,9	1,33
17	62	M	4,0	1	1	20	1	Right	0	0	8,2	1,39
18	59	M	7,0	2	1	32	3	Left	1	3	9,9	1,65
19	58	M	6	2	1	33	5	Right	0	2	11,3	1,25
20	54	M	4,5	2	1	25	2	Left	1	14	13,8	1,32
**Mean**	**63,3**		**5,21**	**1,95**		**27,55**	**2,95**			**4**	**11**	**1,23**

M: Male; F: Female; H&Y: Hoehn & Yahr; FOG: 1) freezer; 0) non freezer; UPDRS: Unified Parkinson’s Disease Rating Scale; PIGD: Postural Instability and Gait Difficulty; Fall risk: 1) yes, 0) no; mFES: Modified Falls Efficacy Scale; TUG; Timed-Up-and-go-Test; TMT: Ten Meter Walk test.

**Table 2:** Participant characteristics.

	Patients	Freezers	Non-freezers	Controls	Group differences
N	20	9	11	9	–
Age	63.3 (8.35)	61.27 (4.84)	64.97 (10.34)	64.67 (5.24)	0.33
Women (%)	30	11	46	22	0.69
Disease duration (years)	5.21 (3.11)	6.81 (3.77)	4.05 (2)	–	0.18
H&Y stage (1 |2|3)	3 | 15 | 3	1 | 6 | 3	2 | 9 | 0	–	0.27
UPDRS III	27.55 (10.44)	31.33 (12.05)	24.45 (8.20)	–	0.65†
Left clinical asymmetry (%)	65	44	82	–	0.08
Clinical asymmetry score	6.3 (3.9)	7.4 (4.4)	5.7 (3.5)	–	0.35
FAB	16 (2.46)	15.44 (2.07)	15.64 (2.84)	–	0.99
NFoG-Q (max 24)	–	12.78 (3.99)	0 (0)	–	<0.0001

Data reflect means (standard deviation between brackets). N; number of subjects, NS; not significant, UPDRS; Unified Parkinson’s Disease Rating Scale, L: Left side most affected H&Y Hoehn & Yahr, FAB; Frontal Assessment Battery. NFoG-Q; new freezing of gait questionnaire.

† Mann-Whitney U test.

Patients were assessed during the OFF state. There were no significant differences between patients and controls, or between freezers and non-freezers.

We excluded patients with marked cognitive dysfunction (Mini Mental State Examination <24 or Frontal Assessment Battery <13 [32]–[34], or with visual, vestibular, orthopaedic, psychiatric, or other neurological diseases. Also, participants with a history of joint injuries were excluded.

### Ethics statement

The research protocol was approved by the medical ethics committee of the local hospital (Medical Spectrum Twente; MST), in accordance with the Declaration of Helsinki. All participants gave prior written informed consent. The PD patients were selected by a movement disorders neurologist (J.P.P. van Vugt), based on the UK Brain Bank criteria.

### Apparatus and recording

**Figure 1:**
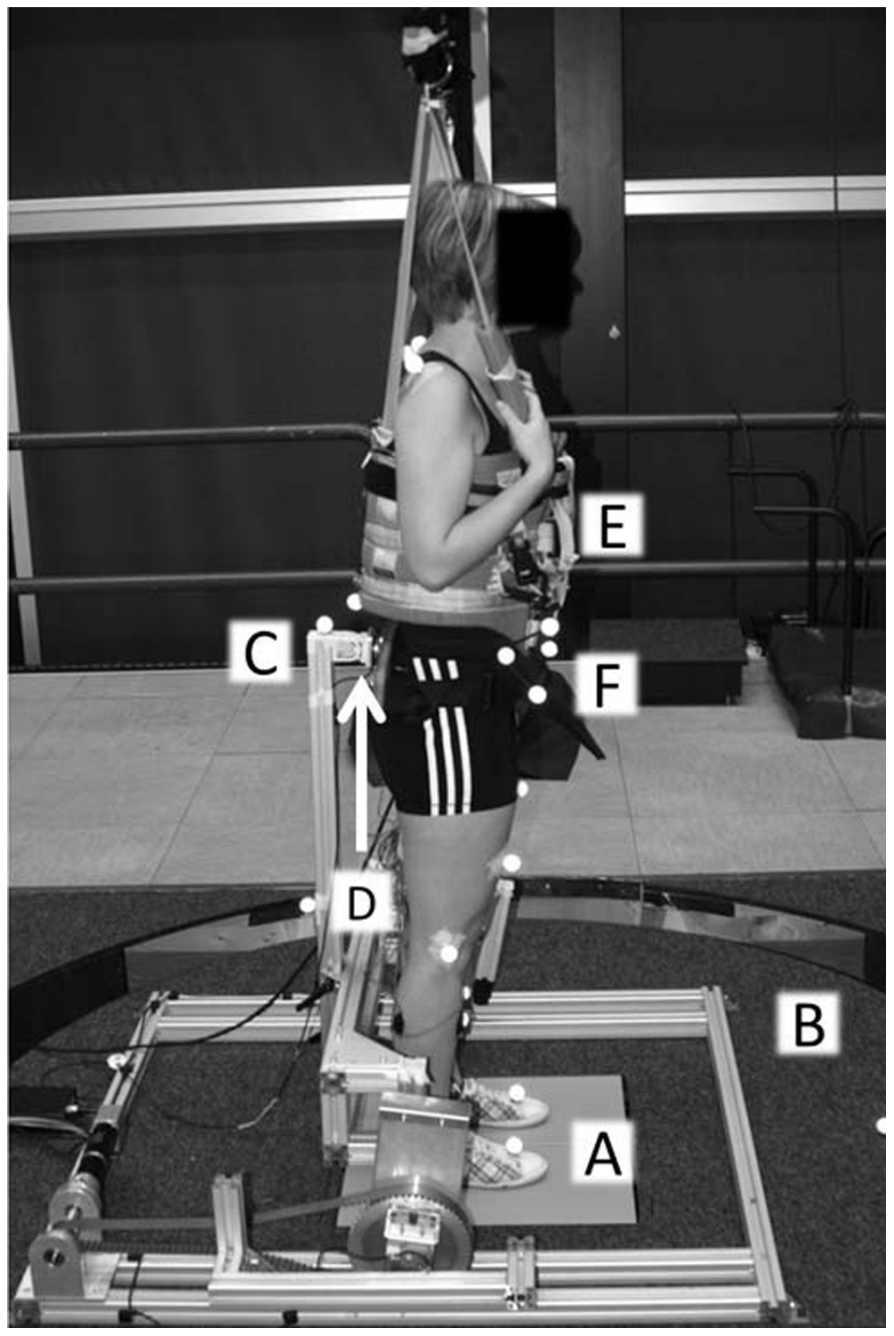
Experimental set-up. Participants stood on the dual forceplate (A), with their arms crossed over their chest, embedded in the movable platform (B). Two independent perturbations in the forward-backward direction were applied simultaneously using both the movable platform (B) and the pusher (C). Interaction forces between the pusher (C) and the participant were measured with a force sensor (D). Actual falls were prevented by the safety harness (E), which did not provide any support or directional cues. Reflective spherical markers (F) measured movements of the participant.

Two independent perturbations were administered with a computer-controlled six-degrees-of-freedom motion platform (Caren, Motek, Amsterdam, The Netherlands) and a custom-built actuated device that was able to apply perturbing forces at the sacrum, called the pusher (Figure 1). The data presented here are part of a larger dataset that also enables the investigation of multi-segmental balance control, i.e. the identification of interactions between the ankle and hip joints [24]. Therefore two perturbations were applied [24], [35], although for the specific research question in this present paper, this is not strictly necessary.

Body kinematics and platform movements were measured using motion capture (Vicon Oxford Metrics, Oxford, UK) at a sample frequency of 120 Hz. Reflective spherical markers were attached to the first metatarsal, calcaneus, medial malleolus, the sacrum, the manubrium, and the last vertebrae of the cervical spine (C7). A cluster of three markers was attached to the anterior superior iliac spines on the pelvis. One additional marker was attached to the foot and two markers were attached to the lower leg (one on the tibia) to improve the estimation of the rotational axis of the ankle joint. Also, markers were attached on the knee (just below the lateral epicondyle) and shoulder joints (just in front of the acromion). Furthermore, three markers were attached to the platform. Reactive forces from both feet were measured with a dual forceplate (AMTI, Watertown, USA), embedded in the motion platform. The signals from the dual forceplate, the six degrees-of-freedom force transducer, and the perturbation by the pusher were sampled at 600 Hz and stored for further processing.

### Disturbance signals

**Figure 2:**
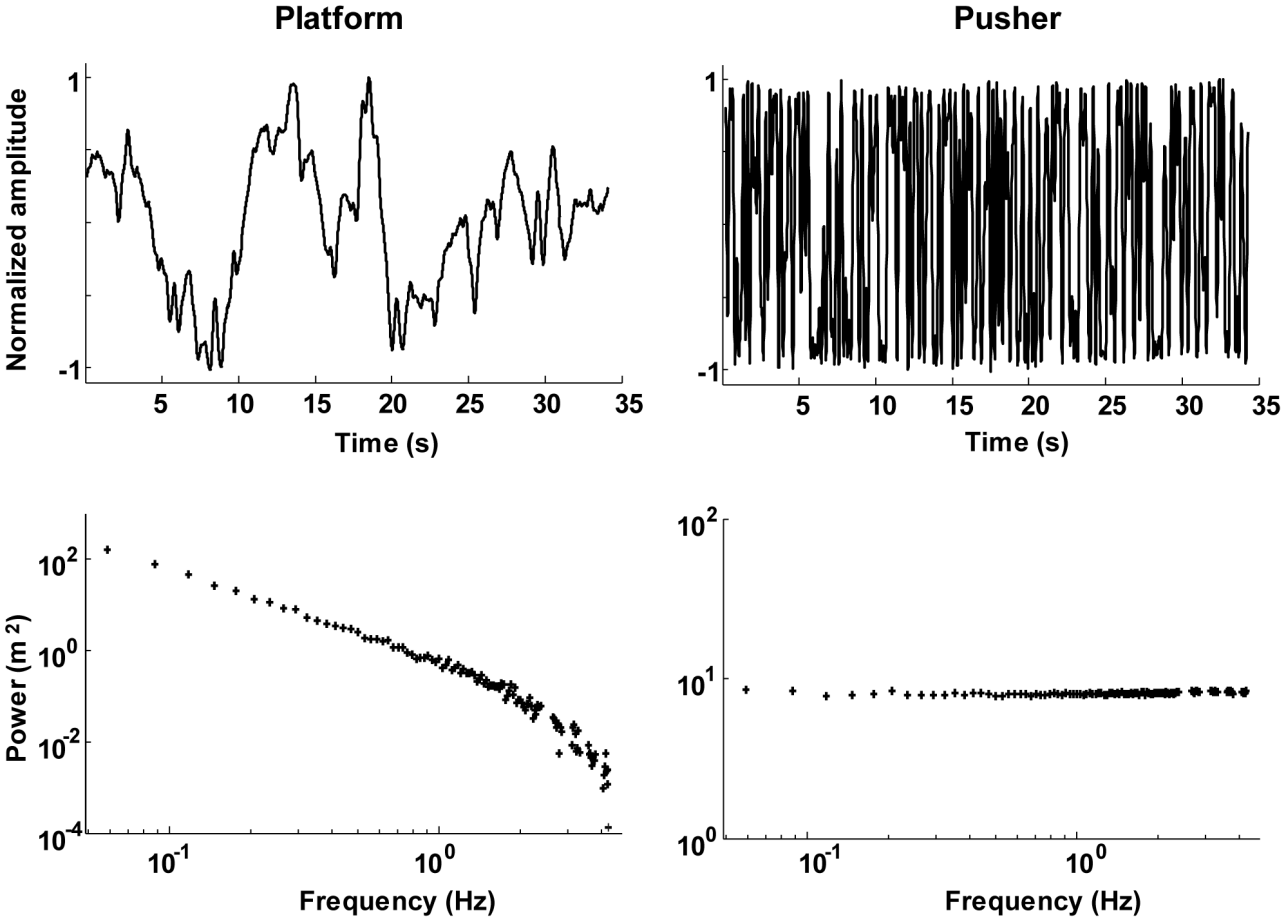
Timeseries and powerspectrum of the two perturbation signals. The left panels represent the platform perturbation; the right panels the pusher perturbation.

The perturbation signal was a multisine with a period of 34.13 sec, previously used in other studies [4], [23], [36], see Figure 2. A multisine has the advantage that it is unpredictable for participants, because the signal consists of many sinusoids [37]. The signal contained power at 112 frequencies (with a frequency resolution of 0.03 Hz) in the range of 0.06–4.25 Hz. This was done because humans respond differently to fast (high-frequency) compared to slow (low-frequency) perturbations [21]. In this way, we could characterize the balance-control behavior from 0.06 to 4.25 Hz.

To increase the power at the excited frequencies, the signal was divided in five frequency bands: 0.06–2.37 Hz (i.e., 80 frequencies; 0.06 Hz, 0.09 Hz, 0.012 Hz etc.), 2.63–2.84 Hz (8 frequencies), 3.11–3.31 Hz (8 frequencies), 3.57–3.78 Hz (8 frequencies) and 4.04–4.25 Hz (8 frequencies); see also Figure 2. Frequency points outside these frequency bands were not excited. The phases of the sinusoids were optimized with crest optimization [35], such that the variance of the signal is maximal at a given amplitude. The perturbation signal was used for both the platform (scaled by the inverse of the frequency) and the pusher (not scaled), see also Figure 2. This procedure resulted in a higher perturbation power at the lower frequencies, compared to the higher frequencies. This was done for two reasons: Balance control is dominated by slow corrective movements [38], which correspond to low frequencies. Secondly, the amount of system noise is the largest at the lower frequencies [36], [39].

We used this specific signal (with its bandwidth and frequency content), as it provokes a consistent response in human subjects [36]. In this way, we increased the reliability of the estimated stabilizing mechanisms [26].

### Procedure

During the experiment, participants stood with their eyes open and with their arms folded in front of their chest on the dual forceplate while being attached with a band strap that opened with a click buckle to the pusher. Heel-to-heel distance was fixed at 14 cm, and the feet contours were taped to ensure the same foot position across trials. Participants were instructed to maintain their balance without moving their feet, while continuous, multisine platform movement and continuous, multisine force perturbations were applied simultaneously in the forward-backward direction (see “Disturbance signals”). Participants wore a safety harness to prevent falling, but it did not constrain movements or provide support or orientation information in any way. Before any data were recorded, the participants were familiarized with the perturbations. The experimenter determined the maximal amplitude that each participant could withstand while keeping their feet flat on the floor, and assessed whether the participant could withstand this amplitude for the total number of trials. We aimed to use as large as possible perturbation amplitudes for each individual participant, to optimize the ratio between external and internal destabilizing torques, thereby increasing the reliability of the estimated stabilizing mechanisms [26].

The experiment started with a static trial; participants stood quietly for 10 sec with their arms folded in front of their chest. Subsequently, four perturbation trials of 180 s each were recorded. If needed, the participants were allowed to rest in between trials.

### Data analysis

Data obtained during the first two trials were analyzed. From the recorded movement trajectories of the markers, the position of the center-of-mass of the predefined segments (i.e., feet, legs, and the HAT) and of the whole body (CoM) were estimated by first calculating the separate positions and rotations of the body segments [40], [41]. Specifically, with regression equations, the mass, CoM position and the inertia tensor moment of the predefined segments (i.e., feet, legs and HAT) and the joint positions were determined. Subsequently, the CoM was determined as the weighted sum of the separate segment positions [41]. From the static trial, the average distance in the sagittal plane from the ankle to the total body CoM (i.e., the length of the pendulum (l_CoM_) was determined. The sway angle was calculated from l_CoM_ and the horizontal distance from the CoM to the mean position of the ankles. Forces and torques of the force plate and force sensor were filtered with a fourth-order low-pass Butterworth filter with a cut-off frequency of 8 Hz and subsequently resampled to 120 Hz. Forces and torques of the force plate were corrected for the inertia and mass of the top cover [42]. On the basis of the corrected forces and torques and recorded body kinematics, ankle and hip joint torques were calculated with inverse dynamics [41]. In addition, the applied platform perturbation was reconstructed from the platform markers.

### Frequency response functions

The time series of the perturbations, sway angle, and the ankle and hip joint torques were separated into data blocks of 34.14 s (i.e., the length of the perturbation signal). Data blocks with missing markers or with unwanted movements such as a step or weight shifting were excluded from further analysis. In this way it was ensured no actual freezing episodes were recorded during the balance task. Furthermore, our method assumes time-invariant and linear behaviour and therefore we have discarded the first response cycle. This resulted in on average seven whole perturbation cycles per participant for the balance-control asymmetry estimation (i.e., on average, one cycle per participant was discarded). Subsequently, the responses were Fourier transformed at the 112 frequencies of the perturbation signal using the fast Fourier transform in Matlab. These were averaged over the cycles to obtain the average individual response, and the average Fourier coefficients of the platform perturbation and the responses (sway angle and left and right joint torques) were used to calculate the power spectral density (PSD) and cross spectral densities (CSDs) between the average Fourier coefficients of the platform perturbation and the sway angle and between the platform perturbation and the left and right joint torques were determined. The PSDs and the CSDs of the responses were then smoothed by averaging over four adjacent frequency points [43]. Lastly, the Frequency Response Function (FRF) of the stabilizing mechanism was estimated with a SISO joint-input-joint-output system-identification technique [23], [26].

An FRF captures the amount and timing of the response of the participant. As such, the gain of the FRF of the stabilizing mechanisms represents how much torque is exerted in response to body sway. The phase gives information about the timing of the response. If there is a phase lead, the response of the joint advances the body movement and an increasing phase lag for example indicates a neural time delay [44].

The FRFs were calculated from sway angle to left and right ankle and hip torques separately. Furthermore, the FRFs were normalized by the mass and length of the participants to compensate for differences in the subjects’ mass and pendulum length, which influence the FRF [44].

### Balance asymmetries

We calculated three balance proportions of both legs, of the weight-bearing and the balance- control contribution of both the ankle and the hip joint separately. The dynamic weight-bearing proportion (DWB) was calculated by calculating the relative weight bearing on the left and right leg as determined from the dual forceplate:
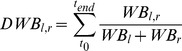
(1)With *t*_0_ and *t*_end_ the first and last sample of the trial.

To determine the relative contribution of each ankle and hip joint to the total amount of generated corrective torque to resist the perturbations, the contribution of the gain and phase of the FRFs of each leg to the gain of the total body was calculated [23]). Subsequently, the contributions were averaged over the frequencies of the perturbation signal to obtain the dynamic balance contribution (DBC) for each leg:
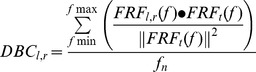
(2)With FRF_l, r_ the left or right FRF and FRF_t_ the total FRF. *F*_max_ is the highest frequency in the signal, *f*_min_ the lowest and *f*_n_ is the amount of frequencies in the signal (i.e., 112). The • indicates the dot product of the FRFs. This resulted in a DBC_Ank_ and DBC_Hip_ for the left and right leg. In this way, the contribution of the left or right leg to the total balance control was expressed as a proportion.

For example, a DBC of 0.8 means that one leg contributed for 80 percent to upright stance, while the other contributed for 20 percent. In order to compare the amount of asymmetry between the non-freezers and freezers, the absolute amount of asymmetry was determined by: |DBC–0.5|. Note that we separated *weight-bearing* asymmetries from *control* asymmetries during upright perturbed stance in this study.

### Statistical analysis

Based on the weight-bearing and balance-control contribution values of the healthy controls, the 99-percent confidence interval (CI) for the weight bearing (DWB), for the ankle (DBC_Ank_) and the hip joint (DBC_Hip_) were determined. Patients whose balance contributions were outside this confidence interval were classified as having asymmetrical balance control.

The patients with asymmetrical balance control (PDASYM) were compared to their symmetrical counterparts (PDSYM). This comparison was made for age, UPDRS and PIGD score, fear of falling, 10 m walk and TUG test, using independent t-test.

In case of non-normal distribution of the data, Mann-Whitney U tests were applied. Gender, H&Y, occurrence of FoG and prior falls were compared with a χ^2^ test. These comparisons were made separately for WB, DBC_Ank_ and DBC_Hip_. In addition, the absolute amount of balance-control asymmetry was compared with independent t-tests, between freezers and non-freezers. Alpha was set at 0.05 and to correct for multiple comparisons, the confidence level was adjusted with Bonferroni correction.

As a secondary analysis, we determined whether the amount of weight bearing (asymmetry) was related to the amount of balance control (asymmetry) by plotting the DBC_Ank_ values against the WB values of the left leg of each individual patient [23]. Subsequently, we fitted a linear regression line between DBC_Ank_ (dependent variable) and WB (independent variable) of the left leg for all PD patients. Subsequently, we compared the slopes (gradient and offset) of the regression lines with a multiple linear regression between DBC_Ank_ and WB for PD patients, with WB as independent variable and DBC_Ank_, freezing and the interaction between freezing and weight bearing as dependent variables. Furthermore, we calculated this regression for freezers and non-freezers separately.

For all statistical analysis we used IBM SPSS statistics, version 20.0.

## Results

**Figure 3:**
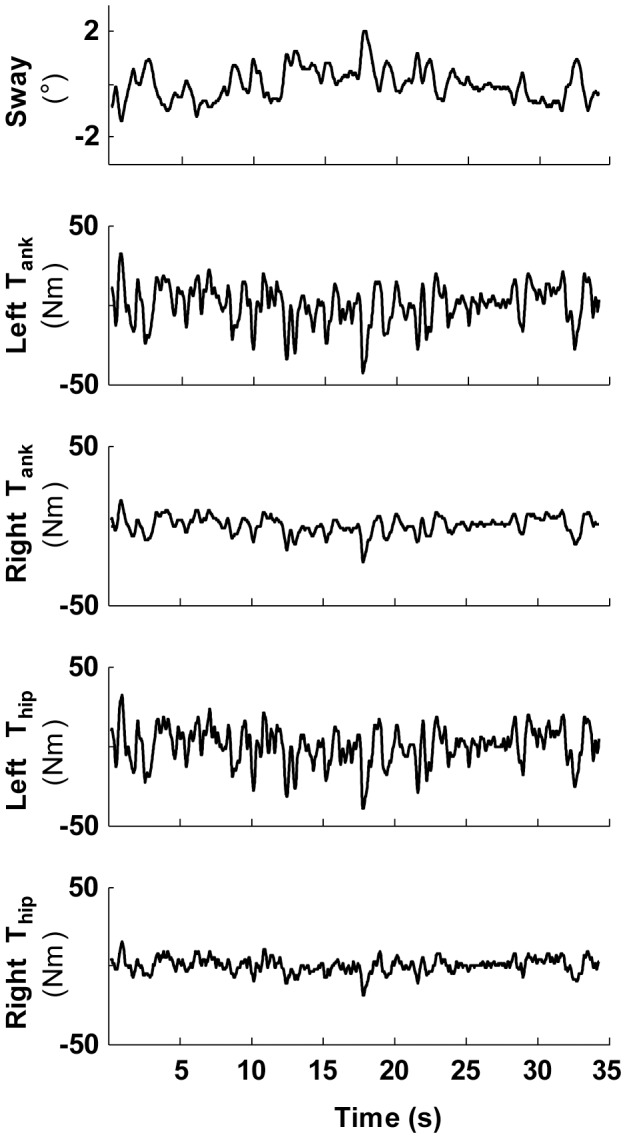
Timeseries of a representative Parkinson patient of one perturbation cycle. The upper two panels represent the platform and pusher perturbations. The lower four panels show the participants’ response. Note that there are clear asymmetries in the joint torque responses.

Both patients and controls were able to maintain their balance in the face of the two applied perturbations in the anterior-posterior direction. The average amplitudes for the platform were similar for controls (0.028 m, std: 0.002) and patients (0.028 m, std: 0.004; t_(25)_ = 0.6, *p* = 0.68). The average amplitudes of the pusher were higher for healthy controls (9 Nm, std: 1) compared to the patients (7.8 Nm, std: 1.56; t_(25)_ = 2.4, *p* = 0.04), see also Figure 3. Controls and patients swayed just as much (mean RMS PD: 0.71°, std: 0.11; HC: 0.74°, std: 0.08; t_(177)_ = 2.98, *p* = 0.13) in response to the perturbations.

### Asymmetries in weight bearing and balance control

**Figure 4:**
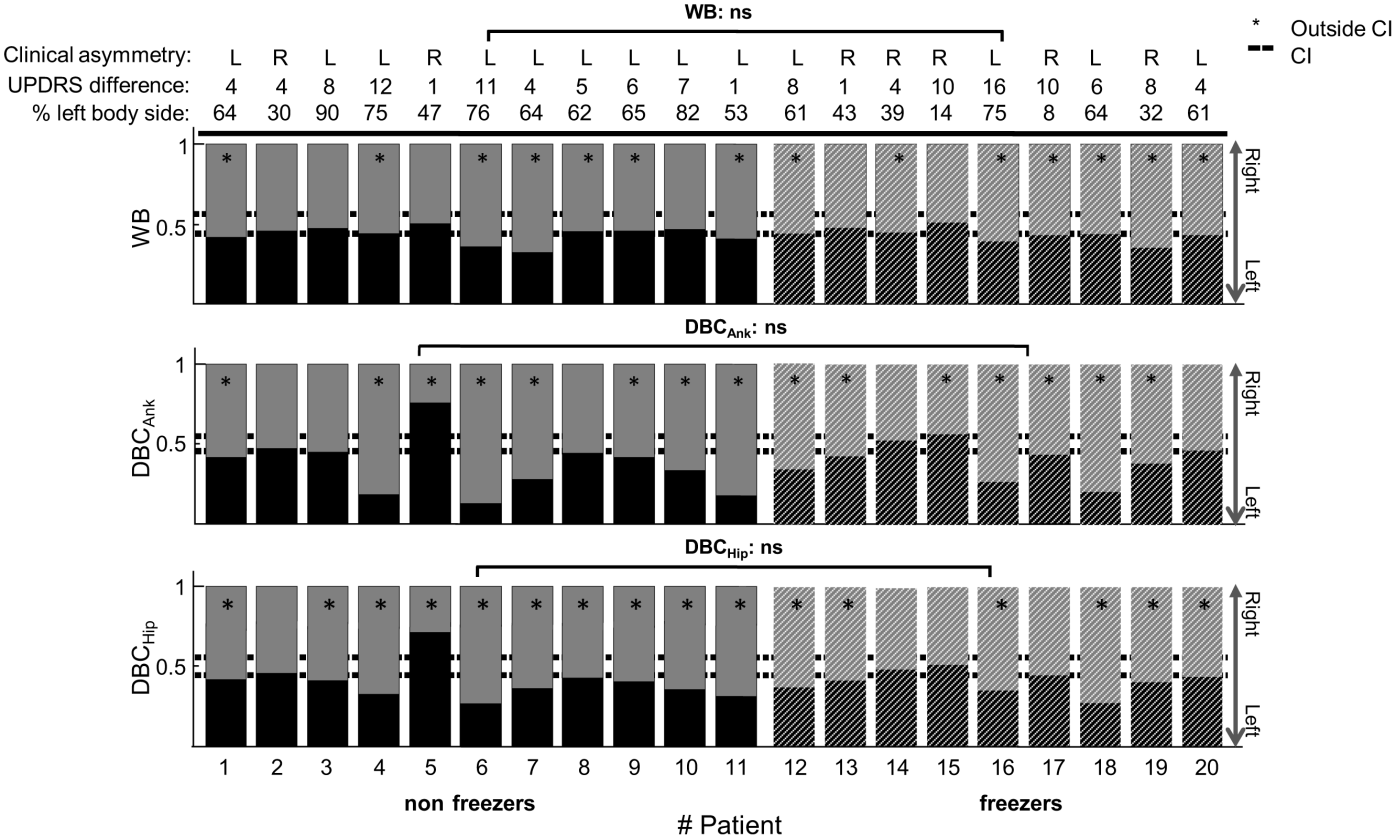
Clinical asymmetry, weight-bearing and balance-control contribution of the left (lower bar) and right leg (upper bar) of the individual PD patients. The absolute and relative value and the most affected side of the clinical asymmetry is shown above the bar graphs. The upper panel indicates the dynamical weight bearing (DWB), the middle panel the dynamic-balance contribution of the ankle joint (DBC_Ank_), and the lower panel is of the hip joint (DBC_Hip_). The group is separated in non-freezers (the first 11 patients, indicated by the solid bars) and freezers (patient 12 through 20, indicated by the dashed bars). There were no significant differences in asymmetry of WB, DBC_Ank_ nor DBC_Hip_ between non-freezers and freezers. The dashed line indicate the 99-percent confidence intervals of the healthy controls for the WB, DBC_Ank,_ and DBC_Hip_. The asterisk (*) denotes balance contributions outside the respective confidence intervals. For WB, 14 patients were outside the 99 percent CI. This number increased to 15 considering DBC_Ank_ and to 16 for DBC_Hip_. ns = not significant. The clinically most affected side coincided in most cases with weight-bearing and balance-control asymmetry. However, there were no significant correlations between clinical asymmetry and balance asymmetry (WB, p = 0.32; DBC_Ank_, p = 0.37 and DBC_Hip_, p = 0.75).

Figure 4 shows the weight-bearing proportion and balance-control contributions (DBC_Ank_ and DBC_Hip_) of the left and right leg of the PD patients. Individual PD patients showed highly asymmetric weight bearing and balance control, whereas healthy controls distributed their weight evenly and exerted equal corrective torques with both legs (mean_WB_: 0.49, std_WB_: 0.056; mean_Ank_: 0.48, std_Ank_: 0.044; mean_Hip_: 0.49, std_Hip_; 0.04; 99% Confidence Intervals (CI) shown in Figure 4). For example, for PD patient six, the left ankle contributed by 13 percent, whereas the right ankle contributed by 87 percent to upright stance. However, there were also PD patients who controlled their balance symmetrically, for example patient two. Although there were balance-control asymmetries at both joints, Figure 4 shows that these were less pronounced at the hip joint. Furthermore, a weight- bearing asymmetry was not always accompanied by a balance-control asymmetry (e.g., pt 5) or vice versa (e.g., pt 13). Also, the amount of weight-bearing asymmetry was not always the same as the balance-control asymmetry. For example, in patients six and 11, the weight-bearing asymmetry was smaller than the balance-control asymmetry of the ankle joint. Most patients used their right leg more than their left leg to maintain upright balance. Except for patient two, all patients showed balance asymmetries, either in unevenly distributed weight or in different balance-control contributions of the left and right leg at the ankle or hip joint.

Furthermore, in most cases, the most affected side as determined clinically (the difference between left and right UPDRS scores) coincided with the balance-control asymmetry as determined with our balance experiment and analysis methods (see Figure 4). However, there were no significant correlations between clinical asymmetry and balance asymmetry (WB: R^2^ = 0.24, p = 0.32; DBC_Ank_: R^2^ = 0.21, p = 0.37 and DBC_Hip_: R^2^ = 0.08, p = 0.75).

### Clinical comparison between PDSYM and PDASYM

When considering weight bearing, 14 of the 20 patients were outside the normative values. This number increased to 15 when assessing balance-control asymmetries at the ankle and to 16 at the hip joint. Subsequently, the clinical characteristics of the symmetrical patients (PDSYM) were compared to the asymmetrical patients (PDASYM) patients based on the normative values of the weight-bearing and the balance-control contribution of the ankle and hip joint (Table 3). In general, patients in the PDASYM group were slightly older and were more likely to be men (except for WB). UPDRS scores, prior falls, fear of falling, walking speed, and turn speed did not significantly differ between both groups. Also, the proportion of freezers was comparable in the PDASYM and PDSYM groups.

**Table 3:** Comparison of clinical outcome measures between patients with (ASYM) and without (SYM) asymmetrical weight bearing or asymmetric balance control, based on the 99 percent CI of weight bearing, the ankle joint or the hip joint contributions of the healthy controls.

	Weight bearing			Ankle joint			Hip joint		
	ASYM_WB_	SYM_WB_	*p*	ASYM_Ank_	SYM_Ank_	*p*	ASYM_Hip_	SYM_Hip_	*p*
N	14	6		15	5		16	4	
Age (yrs.)	63.79 (8.98)	62.5 (6.97)	0.74	63.9 (8)	61.8 (9.9)	0.36	63.63 (8.9)	62.5 (5.8)	0.84
Women (%)	36	17	0.38^∼^	20	60	0.09^∼^	33	50	0.33^∼^
Preferred leg (% Left)	50	33	0.27^∼^	47	40	0.78^∼^	50	25	0.11^∼^
Disease duration (yrs.)	5.29 (3.45)	4.17 (2.8)	0.26	5.2 (2.5)	5.8 (5.2)	0.83	4.25 (2.2)	7.75 (5.5)	0.25
Freezers (%)	50	33	0.49^∼^	47	40	0.79^∼^	38	75	0.18^∼^
H&Y (1 | 2 | 3)	2 | 11 | 1	1 | 4 | 1	0.43^∼^	2 | 11 | 2	1 | 4 | 0	0.67^∼^	2 | 12 | 2	1 | 3 | 0	0.54^∼^
MDS-UPDRS III	31.71 (9.14)	17.83 (5.91)	0.004	29 (10.4)	23 (10.3)	0.36	28.81 (10.5)	22.5 (9.95)	0.27
PIGD	3.57 (1.99)	1.5 (1.76)	0.02	2.93 (1.8)	3 (3.2)	0.72	3.06 (1.69)	2.5 (3.7)	0.25
Prior falls (% with falls)	50	20	0.16^∼^	40	20	0.69^∼^	50	0	0.07^∼^
Fear of falling	4.93 (4.55)	3.17 (3.13)	0.43	3.73 (2.82)	6.4 (6.9)	0.79	4.06 (3.6)	5.75 (6.55)	0.89
TMT (m/s)	1.21 (0.22)	1.29 (0.94)	0.32	1.24 (0.2)	1.22 (0.18)	0.82	1.23 (0.2)	1.21 (0.2)	0.93
TUG (s)	11.15 (2.83)	9.25 (1.93)	0.21	11.05 (2.85)	9.19 (1.65)	0.19	11.07 (2.73)	8.67 (1.57)	0.05

Data reflect means with the standard deviation (between brackets). N, number of subjects, UPDRS Unified Parkinson’s Disease Rating Scale; H&Y Hoehn & Yahr; PIGD; Postural Instability and Gait Difficulty; TMT; Ten Meter walk Test, TUG; Timed-Up-and-go-Test; ASYM = asymmetrical patients. SYM = symmetrical patients.

Due the small sample we used non-parametric tests (Mann Whitney U test) or χ2 tests indicated with (∼).

*p* values are not corrected for multiple comparisons, but the significance level reduced 0.005 due to Bonferoni correction.

There were no significant differences in clinical outcome measures between patients with and without asymmetrical weight bearing or balance control.

### Comparison of balance asymmetries between freezers and non-freezers

We also compared absolute weight-bearing and balance-control asymmetries between freezers and non-freezers. Freezers did not have a more asymmetric weight distribution (mean: 0.07, std: 0.04) compared to non-freezers (mean: 0.07, std: 0.05; t_(18)_ = −0.20, *p* = 0.85). The mean absolute joint asymmetry was slightly smaller for freezers (DBC_Ank_: 0.12, std: 0.09; DBC_Hip_: 0.09, std: 0.07) than for non-freezers (DBC_Ank_: 0.09, std: 0.07; DBC_Hip_: 0.13, std: 0.06). However, freezers did not control their balance more or less asymmetrically than non-freezers (DBC_Ank_; t_(18)_ = −1.18, *p* = 0.25, DBC_Hip_; t_(18)_ = −1.51, *p* = 0.15).

### Relationship between weight bearing and balance control

**Figure 5:**
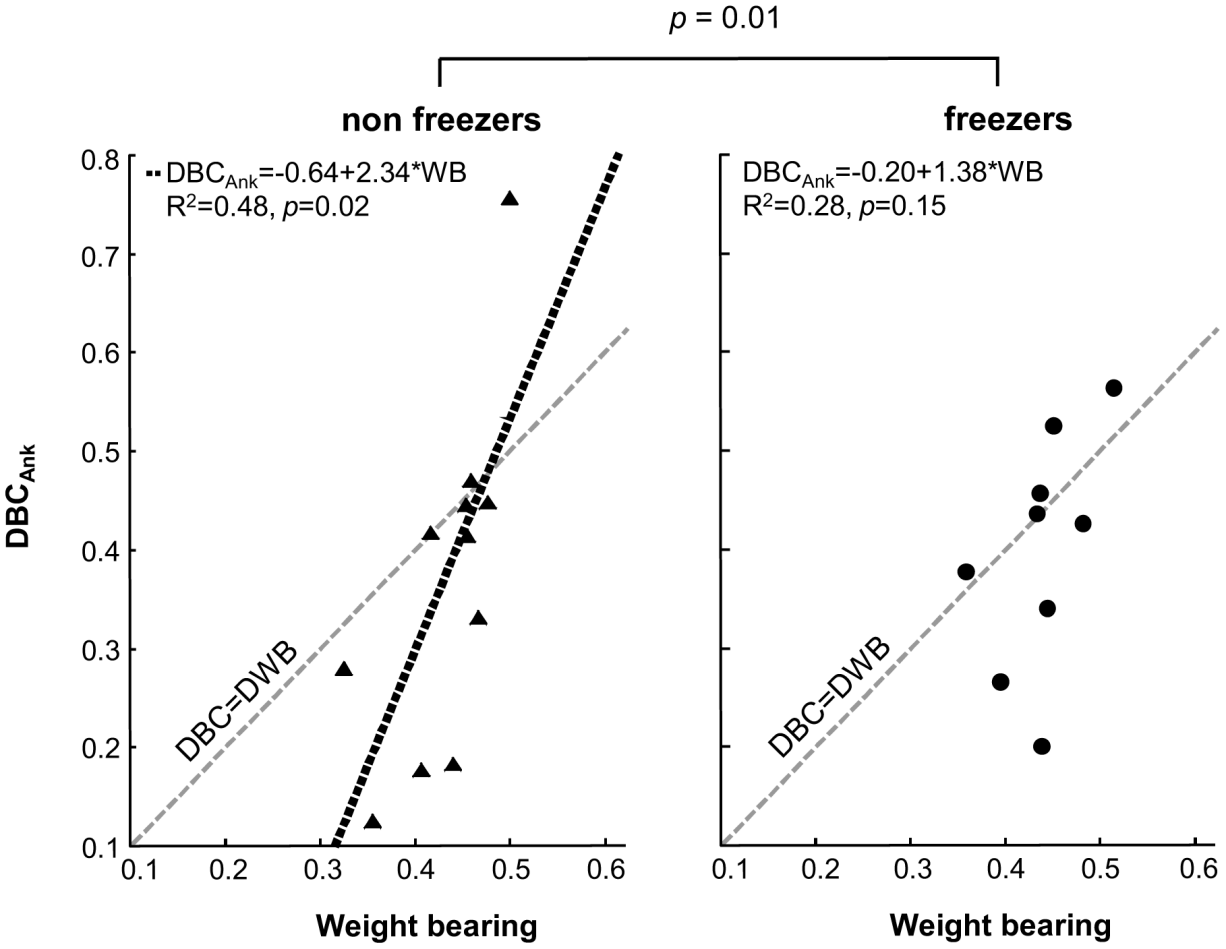
Dynamic-balance contribution of the ankle (DBC_Ank_) vs. weight bearing of PD patients, shown separately for non-freezers (left panel) and freezers (right panel). The healthy one-to-one relationship (DBC = DBW) is indicated by the grey dashed line in both panels. The linear regression line between weight bearing and balance control for the non-freezers is indicated by dotted line. There was a significant difference between regression lines of freezers and non-freezers (*p* = 0.01). Freezers showed a non-significant relationship between weight bearing and balance control (R^2^ = 0.28, *p* = 0.15), whereas non-freezers showed a significant relationship (R^2^ = 0.48, *p* = 0.02). The * indicates a significant difference between the regression lines of the non-freezers and freezers.

As a secondary analysis, we investigated the relationship between weight bearing and balance control by determining the linear regression between DBC_Ank_ and WB of the left leg for PD patients, and separately for freezers and non-freezers (Figure 5). We found a significant linear regression in all PD patients (R^2^ = 0.41; *p* = 0.002), in the non-freezers (R^2^ = 0.48; *p* = 0.02), but a non-significant relationship in freezers (R^2^ = 0.28; *p* = 0.15).

Freezers had a different relationship between weight bearing and balance control compared to non-freezers (t_(19)_ = 2.9, *p* = 0.01). Note that we excluded one data point of the non-freezers for this analysis, as this participant had a unusually high Cook’s value [45]. It should be noted, though, that including this data point resulted in non-significant differences between freezers and non-freezers, but still showed a trend (p = 0.063), indicating that freezers had a weaker relationship between weight bearing and balance control compared to non-freezers.

Similar results were obtained when calculating linear regression between DBC_Hip_ and WB. This means that the (healthy) relationship between weight bearing and balance control is less pronounced in freezers.

One could argue that the difference in the coupling between weight bearing and balance control between freezers and non-freezers is due to the fact that freezers were more severely affected and had a larger variability (see Table 1). Further inspection of the data showed that one of the freezers had a high UPDRS score (in fact this patient’s UPDRS score can be considered an outlier), which increased the variability of the freezers’ UPDRS scores. Excluding this patient from the analysis resulted in different UPDRS scores for the freezers (mean: 28.13; SD: 7.8), but did not alter the main findings of this paper. That is, without this patient there were still no significant differences between freezers and non-freezers for clinical scores. Also, freezers did not have larger asymmetries in balance control compared to non-freezers. The coupling between weight bearing and balance control remained non-significant in freezers (R^2^ = 0.3, *p* = 0.16), and the regression lines of freezers and non-freezers were still significantly different from each other (*p* = 0.02).

## Discussion

The main findings were that most PD patients in our sample showed asymmetries in either weight bearing or in balance control. These balance asymmetries were not related to FoG or other clinical outcomes. However, the normal relationship between weight bearing and balance control was not significant in freezers, but preserved in non-freezers.

### Balance control is asymmetrical in patients with Parkinson’s disease

PD patients used one leg more than the other leg to control their balance. When considering the ankle joint, 15 of the 20 PD patients were outside the normative values. This proportion of patients with asymmetrical balance increased to 16 when separately considering the hip joint. Balance asymmetries in PD patients have been shown before [3]–[5], but this study is the first to investigate balance-control asymmetries in a large group of patients, tested OFF medication, and by applying continuous perturbations in combination with a well-defined model of balance control [44]. As balance control is a closed-loop system, perturbations are necessary to disentangle the control actions from the body mechanics [22], thus separating balance control and weight bearing.

One prior study reported that four out of 17 PD patients (24 percent) showed asymmetrical postural control during quiet stance [5]. In the present study, about 75 percent of patients showed asymmetrical balance control. There are various explanations for this difference in proportion of asymmetrical balance. First, patients in the study of Geurts and colleagues [5] were assessed during quiet unperturbed stance, whereas we perturbed the patients’ balance. As a potential confounding factor, we speculate that the perturbations could have stimulated (partly) unloading of the stepping leg to anticipate a compensatory step, thereby exaggerating a balance-control asymmetry. However, we did control for this by assessing the balance asymmetries of patients during quiet stance (data not shown), and this analysis yielded similar results compared to the dynamic condition reported here. Second, patients were tested ON medication in the study by Geurts and colleagues, whereas we assessed patients OFF medication. The effect of dopaminergic medication on postural control is difficult to predict, as some elements may improve, while others are resistant to medication or even worsen in the ON state [46]–[51]. Indeed, one other study reported that levodopa increased balance asymmetry [3], perhaps because of dyskinesias in two of the six patients during the ON phase. None of the patients in our study showed any discernible dyskinesias, and the CoP traces did not show any random weight shifting. Hence, the present results and those of Geurts and colleagues [5] suggest that depletion of levodopa increases postural asymmetry in patients with PD. However, future studies investigating balance asymmetries ON and OFF medication are needed to confirm this hypothesis.

### Balance control asymmetries are not related to freezing of gait

Our study confirms that asymmetries in weight bearing or balance control are not necessarily present in each individual patient with PD [5]. Our primary interest was to examine whether the presence or severity of balance asymmetries might relate to FoG. This question was driven by the notion that for gait, asymmetries are related to FoG in PD [9], [15], [52]. However, our hypothesis was not confirmed: (a) freezers did not have greater asymmetries in balance control than non-freezers; and (b) freezers were not overrepresented in the group of patients with balance asymmetries. This suggests that motor asymmetries, and specifically corrective balance control asymmetries in the sagittal plane, are not related to FoG. Recent work is actually in accordance with our findings; two recent studies found no differences in asymmetries during gait between freezers and non-freezers [8], [53]. Also, when systematically controlling for step length, no differences in gait asymmetry were found between freezers and non-freezers during the condition where most freezing episodes occurred [52]. In addition, freezing episodes were equally common during turning toward the most or least affected leg [54], again suggesting that motor asymmetry does not play a major role for FoG. Taken together, we feel that other pathophysiological explanations seem more likely, in particular the hypothesis that FoG results from an abnormal *coupling* of balance with gait [11]. However, it could be possible that an impaired regulation of bilateral timing, leading to asymmetric leg swing times [9], [10], did not show up in our balance task.

### Relationship between weight bearing and balance control seems to be disturbed in freezers

In healthy controls, there is a linear relationship between weight bearing and balance control (defined as the exerted corrective joint torque in response to body movement, captured by the dynamic balance control in this work). Specifically, when healthy controls put more weight on one leg, they also use that leg more to produce corrective torques, i.e., they control their balance more with that leg in the anterior-posterior direction [23]. This is reflected by a one-to-one relationship between weight bearing and balance control in healthy controls.

Although preliminary, our results indicate that a relationship between weight bearing and balance control is preserved in PD patients who are non-freezers, although not in a one-to-one fashion as is normally seen in healthy controls [23]. In contrast, was even weaker in freezers, and in fact significantly less than in non-freezers. We do want to stress that we have only investigated the relationship on a group level and future experiments should further investigate this notion in individual patients. In addition, group sizes should be increased to test the robustness of our findings.

There are no other studies that explicitly investigated this relationship. However, one study found that freezers require *multiple* medio-lateral weight shifts before taking a step [55]. In contrast, healthy subjects and non-freezers generate only a *single* lateral weight shift. The authors suggested that these multiple weight shifts in freezers reflected an inability to couple a normal APA to the stepping motor pattern. Our results seem to extend these findings, suggesting that it is not merely the relationship between weight bearing and the stepping motor pattern that is abnormal, but rather that weight bearing and balance control in general are not normally coupled in freezers, also during feet-in-place responses. We hypothesize that this abnormal coupling between weight bearing and balance control could cause FoG episodes: The patient wants to lift the foot, but is unable to automatically shift the body weight towards the stance leg, causing the characteristic feeling of being “glued” to the floor.

### Limitations

We determined the normative values based on the results of only nine healthy controls, which is a relatively small group. However, the postural responses of this control group were very homogeneous, as reflected by the small standard deviations, and they regulated their balance very symmetrically. Therefore, even small balance asymmetries in patients placed them outside the normative values. Consequently, both PD patients with a relatively mild asymmetry and patients with severe asymmetry were classified as abnormal. In addition, based on prior work [5], we expected to find 50 percent of patients with asymmetrical balance control, but instead we found that balance was asymmetric in 75 percent of our sample; this decreased the statistical power for the comparisons between the symmetrical patients and asymmetrical patients.

We included nine freezers (defined as patients who reported the characteristic FOG episodes), but only three of these freezers experienced a FoG episode during the neurological assessments. However, we are confident that the other patients were correctly classified as freezers, as they all reported the typical FoG events during history taking and the scores on the NFOG-Q (including a video with FoG) were high in these patients. FoG is difficult to elicit in an experimental setting, so asking about FoG and using validated questionnaires is often a better indicator for the presence and severity of this phenomenon [11]. In addition, we found a rather large variability in the freezer group, which could have influenced our results. However, the differences and variance between freezers and non-freezers are comparable to other studies that used a similar experimental design [27], [53].

Furthermore, the results of the relationship between weight bearing and balance control need to be interpreted with care, as we compared relatively small groups and the differences were not that large.

### Future perspectives

Future studies should focus on investigating the underlying pathophysiology of balance-control asymmetries and the relationship between weight bearing and balance control. What causes balance-control asymmetries? Are these due to asymmetries in rigidity, or is it perhaps a lateralized proprioceptive problem, which has been suggested to play a role in Pisa syndrome, another example of a postural asymmetry [56]? In addition, it has been shown that PD patients have asymmetries in axial kinesthesia [50] and that levodopa - surprisingly - worsens this. The role of proprioceptive information could be investigated by assessing muscle properties and sensory reweighting capacities of each leg [57]. Also, the effect of levodopa on sensory integration and balance-control asymmetries in general could be tested by assessing patients ON and OFF medication. Furthermore, to study the relationship between weight bearing and balance control in both freezers and non-freezers, patients should be instructed to put a predefined amount of weight on one leg (e.g., 30, 40, and 50 percent of body weight) and subsequently the amount of control exerted with [58] or the quality of a protective step of that leg should be assessed. Also, group sizes should be increased to show the robustness of our findings.

In addition, this study suggests that the relationship between weight bearing and balance control is disturbed in freezers, which could possibly hamper APAs to unload the stepping leg. This weight shift is mainly caused by movements in the medio-lateral plane, and therefore it would be interesting to perturb patients’ balance in the frontal plane. These types of experiments should further clarify the pathophysiology and clinical relevance of postural asymmetries in PD.
